# Imposed positive defocus changes choroidal blood flow in young human subjects

**DOI:** 10.1007/s00417-022-05842-z

**Published:** 2022-09-29

**Authors:** Barbara Swiatczak, Frank Schaeffel, Giacomo Calzetti

**Affiliations:** 1 Institute of Molecular and Clinical Ophthalmology Basel (IOB), Mittlere Strasse 91, 4056 Basel, Switzerland; 2 Section of Neurobiology of the Eye, Ophthalmic Research Institute, University of Tuebingen, Tuebingen, Germany; 3 Zeiss Vision Lab, Ophthalmic Research Institute, University of Tuebingen, Tuebingen, Germany; 4 Department of Ophthalmology, University of Basel, Basel, Switzerland; 5 Department of Ophthalmology, University of Parma, Parma, Italy

**Keywords:** Myopia, Choroid, Blood flow, Defocus, Eye growth, Laser speckle flowgraphy

## Abstract

**Purpose:**

It has previously been found that imposing positive defocus changes axial length and choroidal thickness after only 30 min. In the present study, we investigated whether these changes may result from an altered choroidal blood flow.

**Methods:**

Eighteen young adult subjects watched a movie from a large screen (65 in.) in a dark room at 2 m distance. A 15-min wash-out period was followed by 30 min of watching the movie with a monocular positive defocus (+ 2.5D). Changes in axial length and ocular blood flow were measured before and after the defocus, by using low-coherent interferometer (LS 900, Haag-Streit, Switzerland) and a laser speckle flowgraphy (LSFG) RetFlow unit (Nidek Co., LTD, Japan), respectively. Three regions were analyzed: (1) the macular area, where choroidal blood flow can be measured, (2) the optic nerve head (ONH), and (3) retinal vessel segments.

**Results:**

Changes in choroidal blood flow were significantly and negatively correlated with changes in axial length that followed positive defocus in exposed eyes (*R* =  − 0.67, *p* < 0.01). The absolute values of changes in choroidal blood flow in the defocused eyes were significantly larger than in the fellow control eyes (2.35 ± 2.16 AU vs. 1.37 ± 1.44 AU, respectively, *p* < 0.05). ONH and retinal blood flow were not associated with the induced changes in axial length.

**Conclusions:**

Positive defocus selectively alters choroidal, but not retinal or ONH blood flow in young human subjects after short-term visual exposure. The results suggest that blood flow modulation is involved in the mechanism of choroidal responses to optical defocus.


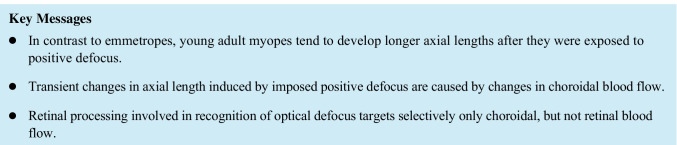


## Introduction

The main function of ocular blood circulation is to maintain homeostasis in the eye which includes thermoregulation and supply of oxygen and nutrients to the ocular tissues. Ocular vessels dynamically regulate blood flow which can rapidly change over time depending on the ocular needs [1]. Changes in fundal blood circulation also represent an important indicator in ocular pathologies. Decreased ocular blood flow can play a role in the development of eye diseases such as chorio-retinal atrophy in high myopia, glaucoma, or age-related macular degeneration [2–6]. On the other hand, an increase in ocular blood flow can be induced in healthy eyes by Ganzfeld or local flickering light which prominently increases retinal neuronal activity [7, 8].

Choroidal blood circulation accounts for 85% of total ocular blood flow [9]. During emmetropization, visual input activates a signaling cascade from the retina through the choroid to the sclera which controls the fine-tuning of axial eye growth rates such that refractive state approaches an optimum in late childhood. Moreover, it is known that process of emmetropization is controlled locally by the retina and involves changes in choroidal thickness [10–14]. It has been shown that the mechanism of emmetropization can be studied in short-term experiments by measuring the miniature changes in axial length and choroidal thickness that are induced by defined visual stimulation (range minutes to hours), both in animal models and human subjects [15–21]. In such experiments, the measured short-term changes in axial length (as determined from the corneal apex to the vitreo-retinal interface) result from changes in choroidal thickness: axial eye shortening based on choroidal thickening and axial elongation based on choroidal thinning [22, 23]. It has been hypothesized by Wallman et al. that changes in choroidal thickness may represent “a third mechanism of focusing the eye” as it can move the photoreceptor plane closer the focal plane [10]. However, such a mechanism would be effective only in small eyes like in chicken where up to 7D of myopia can be rapidly corrected by almost threefold thickening of the choroid [10]. In humans, the amplitudes of choroidal thickness changes account for only fractions of a diopter [17]. Also in monkeys, the optical effects of choroidal thickening are negligible [24, 25]. Therefore, the biological reasons for the observed changes in choroidal thickness may be different. Feldkaemper et al. found that imposed positive defocus in chicken upregulates metabolic energy markers in the retina, like cytochrome-c oxidase [26]. Zhao et al. found that myopia development is accompanied by an increase in hypoxia markers like HIF-1alpha [27]. It was also found that myopia can be inhibited in the guinea pig model by pharmacologically increasing choroidal blood flow [28]. An alternative explanation for choroidal thickening might therefore be that inhibition of eye growth has a high metabolic demand and requires higher choroidal blood supply. It would therefore be of great interest to determine the changes in retinal and choroidal blood flow in response to imposed positive defocus. With moderate amounts of positive defocus, the changes in spatio-temporal features in the retinal image are relatively small. There are almost no changes in retinal luminance but only reduced the steepness of temporal luminance profiles during eye movements spatial due to the optical low-pass filtering [29]. The visual stimuli triggering an increase in choroidal thickness cannot be compared to flickering light which also thickens the choroid [7, 8, 30]. It would be most interesting to find out how positive defocus affects fundal blood flow. We imposed + 2.5 D of optical defocus to one eye in young adult human subjects. The contralateral eye wore the habitual optical correction, if necessary. Short-term changes in choroidal, optic nerve head (ONH) and retinal blood flow, as assessed with laser speckle contrast imaging (LSCI), as well as changes in axial length, were measured after 30 min of exposure.

## Materials and methods

Eighteen healthy young adult participants took part in the experiment (measurements of choroidal blood flow: n = 18, average age: 28 ± 3 years, 9 males, average refractive error: OD − 1.03 ± 1.78D in the range -5.25 to 1.70D, OS -1.11 ± 2.01D in the range -6.00 to 2.25D). Due to a poor quality of optic nerve head LSCI, two myopic subjects were excluded from the data analysis of ONH and retinal blood flow (measurements of ONH and retinal blood flow: n = 16, average age: 28 ± 3 years, 8 males, average refractive error: OD -0.57 ± 1.21D in the range − 3.00 to 1.7D, OS − 0.63 ± 1.48D in the range − 4.25 to 2.25D). Noncycloplegic refractions were determined in all participants by a commercial photorefractor (plusoptiX A12R, PlusOptix, Nürnberg, Germany). Refractive errors are presented as spherical equivalents. Subjects exhibiting anisometropia or astigmatism larger than 1D were excluded. None of the participants had any chronic diseases, prior or current retinal or optic nerve pathologies as assessed with a comprehensive ophthalmological examination. Fundus photographs of both eyes were taken from each individual participant and used as a template for further blood flow analysis. The participants were instructed to restrain from smoking and coffee drinking 2 h before the experiment. All measurements were performed in the morning, between 9 and 11 AM. The study was conducted in accordance with the tenets of the Declaration of Helsinki and approved by the Swiss Research Ethics Committee (EKNZ, reference 2020–01,576). Written informed consent was obtained from each subject prior the experiments.

**Fig. 1:**
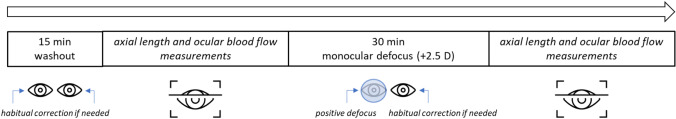
Experimental protocol. The washout period was followed by 30 min of watching a movie with imposed monocular positive defocus (+ 2.5 D). Axial length and blood flow were measured in both eyes before and after the defocus period

Participants watched a movie on a large TV screen (LG OLED65C9, 65 inch, 4 K, 2019, screen luminance ranging 100–300 cd/m^2^) at 2 m distance in a dark room (TV screen was the only light source). The 2 m distance assured that accommodation was in relaxed state (< 0.5D). First 15 min of a washout period was implemented to remove possible influences of previous visual experience on ocular biometry or blood pressure. During this time, participants watched a movie with their habitual distance correction, if needed. Next, the washout period was followed by 30 min of watching a movie with a monocular (right eye) positive defocus imposed by a trial lens (+ 2.5 D) (Fig. 1). Left eye wore habitual distance corrections (if needed) during the entire experiment and served as a control. Before and after exposure to positive defocus, axial length and ocular blood flow were measured in both, control and defocused, eyes.

Axial length was measured as the distance between corneal apex and vitreo-retinal interface by a commercial low coherence interferometer, the Lenstar (Lenstar LS 900 with autopositioning system; Haag-Streit, Koeniz, Switzerland). Five repeated measurements were taken for each data point, before and immediately after the visual stimulation period. Standard deviations of repeated measurements in all subjects were, on average, 6 ± 2 µm.

**Fig. 2:**
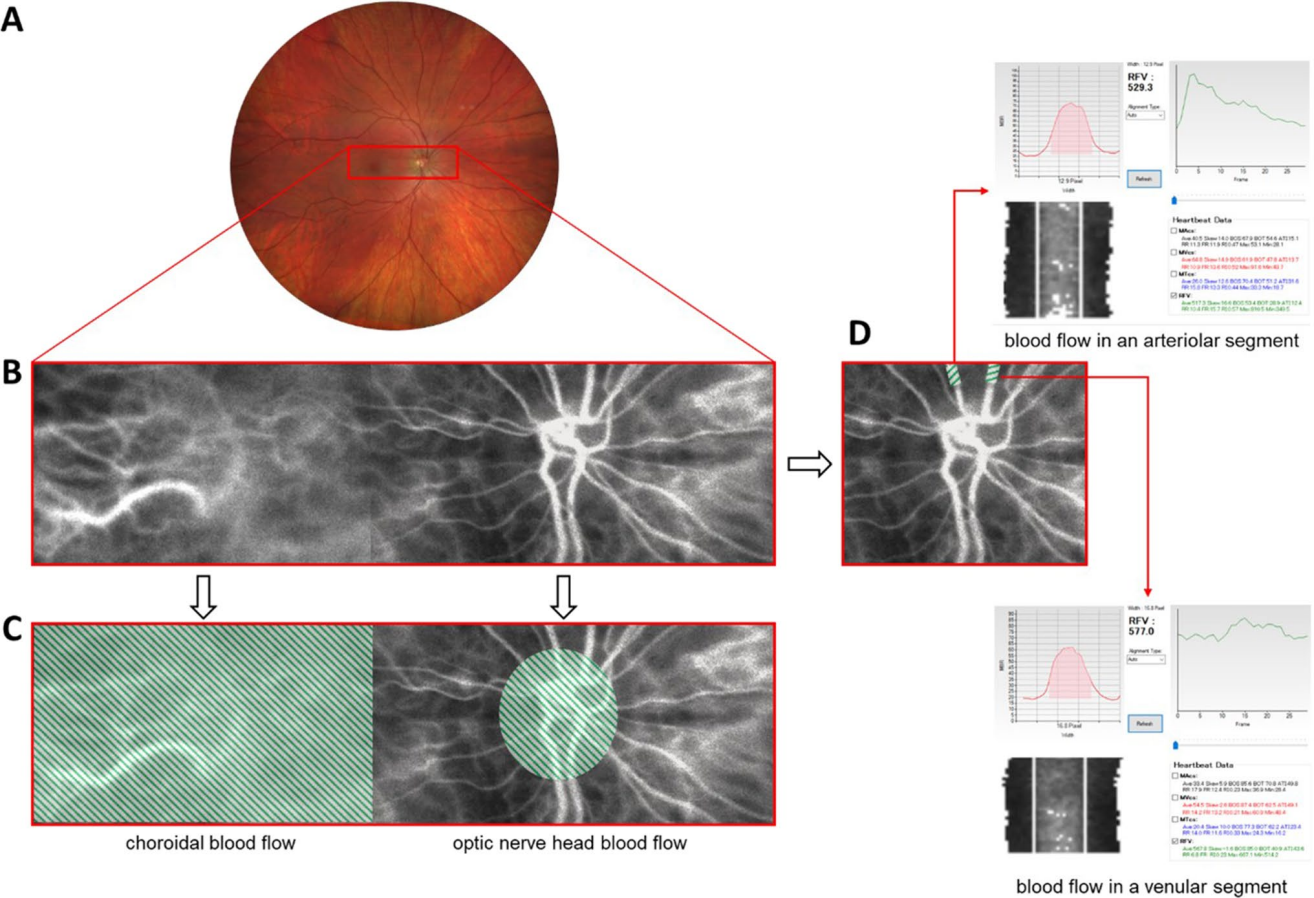
LSFG Analysis. (**A**) Color fundus photograph used as a reference to mark optic nerve head (ONH) borders and to discriminate between retinal arteries and veins. (**B**) Example of a LSFG scan displayed as a “Composite Map”. (**C**) Region of interests (marked in green) for choroidal (left) and ONH (right) blood flow analysis. (**D**) Example of the retinal blood flow measurement in an arteriolar segment (top) and in a venular segment (bottom) of the peripapillary region

The ophthalmic application of LSCI technique has been previously described in detail by Sugiyama [31]. Briefly, the speckle pattern represents a random interference effect that is observed when a laser light is reflected or scattered from a surface such as the ocular fundus. This speckle pattern varies rapidly over time when the illuminated object moves, like the erythrocytes in the ocular vessels. These fluctuations cause a reduction in the contrast of the speckle pattern and provide information about the movement velocity of cellular blood components [32]. In the present study, ocular blood flow was measured by using a commercially available Laser Speckle Flowgraphy (LSFG) system (RetFlow; Nidek Co. Ltd., Gamagori Aichi, Japan) consisting of a fundus camera supplied with an 830-nm diode laser and a digital charge-coupled device camera (750 × 360 pixels). The device records the speckle pattern contrast for approximately 4 s at a frequency of 30 frames per second, providing as an output the “Mean Blur Rate” (MBR), expressed in arbitrary units (AU). The MBR averaged over the 118 frames of an individual LSFG recording is displayed in a grayscale (or pseudocolor) “Composite Map” (Fig. 2B). The MBR has been shown to be highly correlated with volumetric blood flow rates in the optic disk of animal models, irrespective of fundus pigmentation and optic disk atrophy [33–35]. Recently, the Relative Flow Volume (RFV) parameter has been introduced to isolate blood flow of select retinal vessel segments [36]. High reproducibility has been previously reported for LSFG in healthy and diseased human eyes [36–40].

In the present study, the following parameters were investigated as follows: (1) macular MBR, (2) ONH MBR, and (3) retinal RFV (Fig. 2). Two consecutive LSFG scans centered at the optic disk and two centered at the fovea before and shortly after visual stimulation were obtained. All scans passed an in-built quality check as provided by the vendor. Additionally, all scans were controlled for artifacts (e.g., vitreous floaters overlying the region of interest) by an experienced operator. Fovea centered scans were used to analyze macular MBR, while optic disk centered LSFG scans were used to analyze ONH MBR and retinal RFV, as previously described [31, 36, 41, 42]. The macular MBR was used as a measure of choroidal blood flow based on previous experiments showing that MBR as assessed in a macular region including both retinal and choroidal circulation is 92% a measure of choroidal circulation [41]. For the analysis of macular MBR (henceforth referred to as choroidal blood flow), the entire field of the LSFG image was included within a rectangular region of interest, whereas custom elliptical and rectangular regions of interest were drawn for ONH and retinal blood flow, respectively (Fig. 2C, D). A color fundus photograph was available as a reference to precise marking boarders of the ONH and to distinguish retinal arteries from retinal veins (Fig. 2A) [42]. The average MBR within the region of interest was calculated for both choroidal and ONH blood flow analysis, whereas retinal blood flow was analyzed by means of the RFV parameter in one arteriolar and one venular retinal vessel segments located in the peripapillary region (Fig. 2D). All analyses were performed by using the LSFG Analyzer software (version: 3.8.0.4; SoftCare, Fukuoka, Japan) [36].

All statistical analyses were performed using a freely available software for statistical computing “R” (version R 4.0.1; R Core Team (2020), R: A language and environment for statistical computing. R Foundation for Statistical Computing, Vienna, Austria, https://www.R-project.org/). Normal distribution of the data was confirmed with the Shapiro–Wilk test. Effect of positive defocus on axial length was calculated using the paired Student’s *t*-test. Two consecutive LSFG scans were used to characterize intrasession reproducibility. The intraclass correlation coefficient (ICC) was calculated using two-way mixed-effects model with 95% confidence intervals and absolute agreement between repeated measurements was presented as Bland–Altman plot and Pearson’s correlation coefficients. The effects of changes in axial length over induced changes in blood flow in all measured regions were evaluated by Pearson’s correlation coefficient. Moreover, the absolute value of those changes was compared between defocused and control eyes by paired Student’s *t*-test. *P*-values lower than 0.05 were approved as significant.

## Results

**Fig. 3:**
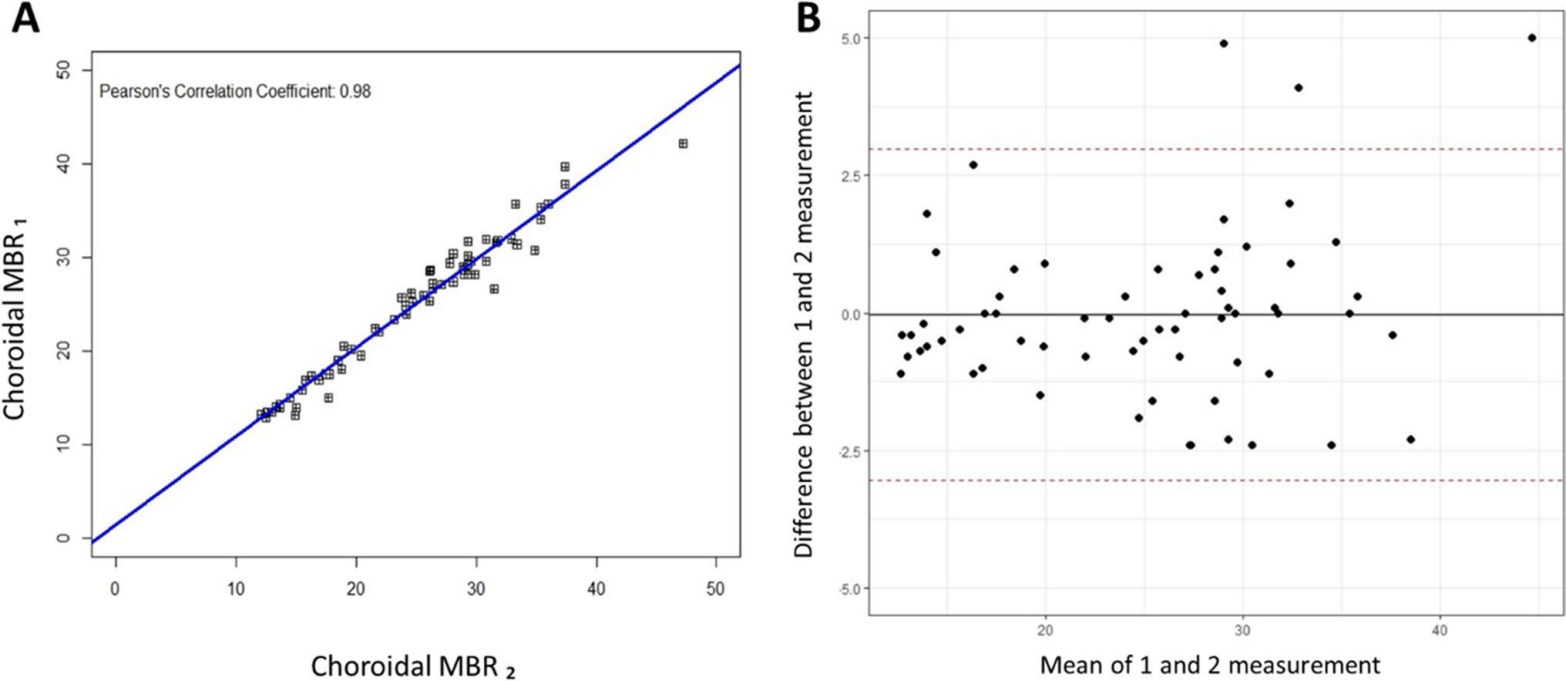
Repeatability of choroidal blood flow measurements. (**A**) Correlation between two consecutive choroidal blood flow measurements from both eyes: MBR_1_ and MBR_2_ (*R* = 0.98, ICC = 0.99, *p* < 0.0001). (**B**) Bland–Altman Plot representing two repeated measurements of choroidal blood flow. Red dashed lines represent 95% confidence intervals

**Fig. 4:**
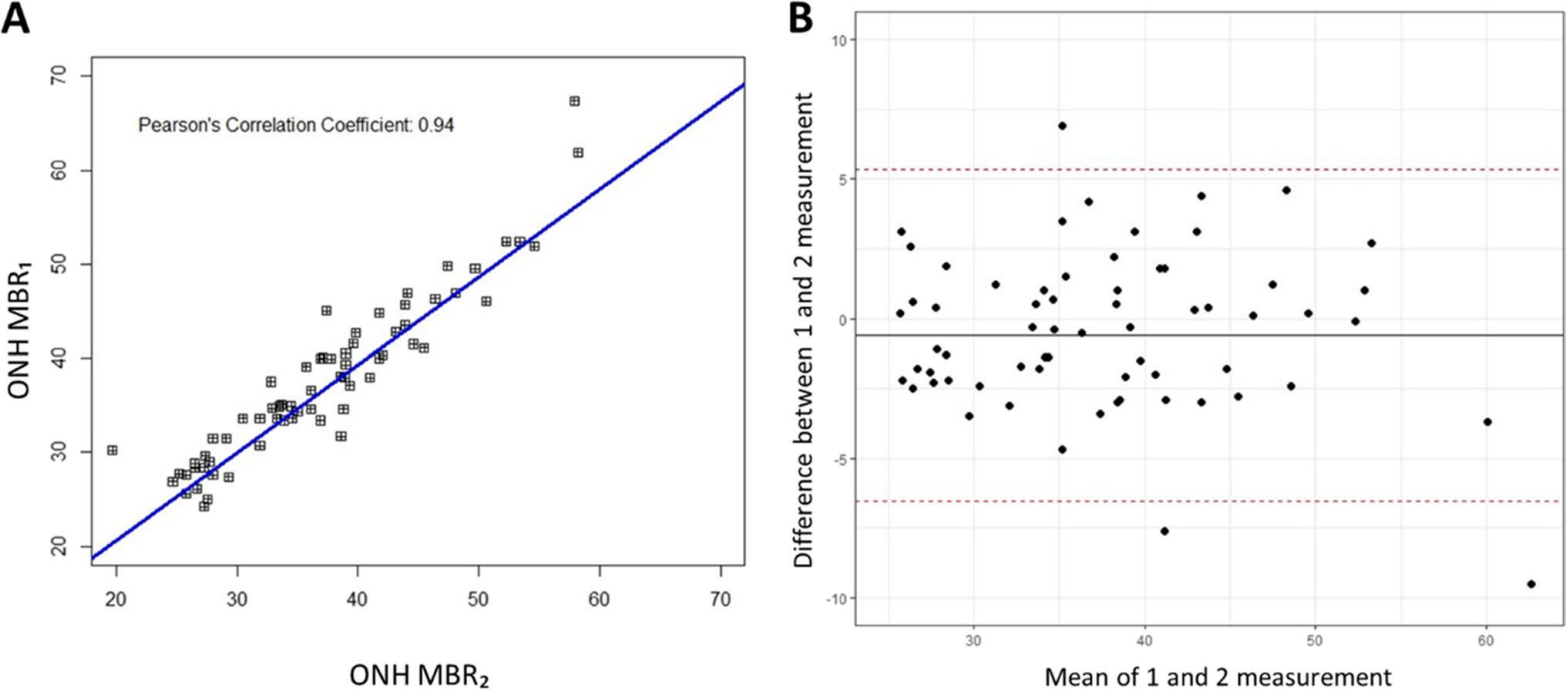
Repeatability of optic nerve head (ONH) blood flow measurements. (**A**) Correlation between two consecutive ONH blood flow measurements from both eyes: ONH MBR_1_ and ONH MBR_2_ (*R* = 0.94, ICC = 0.97, *p* < 0.0001). (**B**) Bland–Altman Plot representing two repeated measurements of ONH blood flow. Red dashed lines represent 95% confidence intervals

**Fig. 5:**
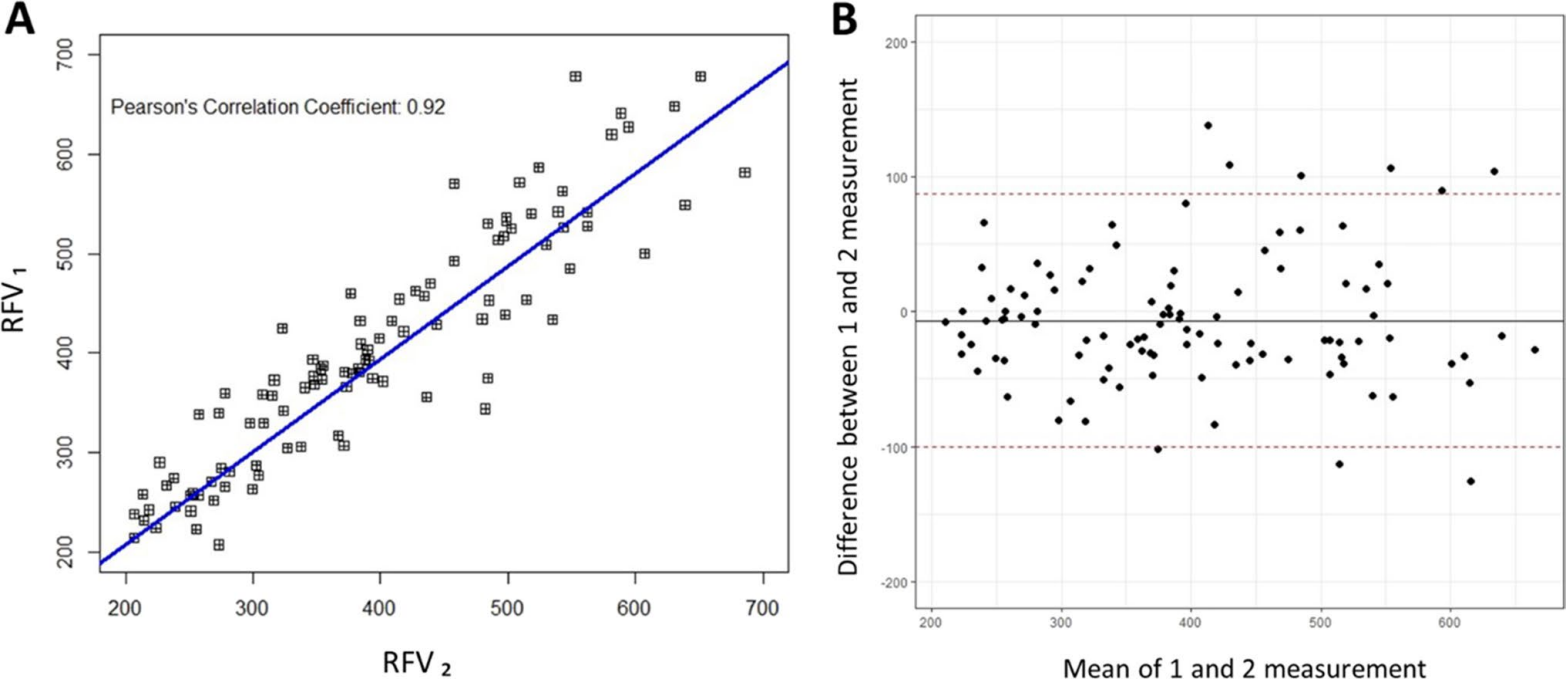
Repeatability of retinal blood flow measurements. (**A**) Correlation between two consecutive retinal blood flow measurements from both eyes: RFV_1_ and RFV_2_ (*R* = 0.92, ICC = 0.96, *p* < 0.0001). (**B**) Bland–Altman Plot representing two repeated measurements of retinal blood flow. Red dashed lines represent 95% confidence intervals

Two consecutive LSFG scans were taken at each measurement point (before and after the defocus). Repeatability of LSFG measurements was calculated separately for three regions of interests as follows: (1) the macula – choroidal MBR_1_ and choroidal MBR_2_, (2) optic nerve head – ONH MBR_1_ and ONH MBR_2_, and (3) retinal vessels – RFV1 and RFV2. Reliability of blood flow measurements for all regions of interest was excellent (all ICCs above 0.90) [43]. Macular region representing choroidal blood flow had the highest agreement between two consecutive scans, expressed as a Pearson’s correlation coefficient (*R* = 0.98) and intraclass correlation coefficient (ICC = 0.99) (Fig. 3). Repeatability of ONH and retinal blood flow measurements showed a similar trend (*R* = 0.94, ICC = 0.97 and *R* = 0.92, ICC = 0.96, respectively) (Fig. 4 and 5).

### Effects of positive defocus on ocular blood flow

Optical defocus (+ 2.5D) induced significant axial length changes in exposed eyes, comparing with the control eyes. Control eyes slightly elongated over the 30 min period which could be explained by diurnal ocular fluctuation, which has been shown to increase ocular axial length in the morning and decrease it in the evening. Nevertheless, those changes did not reach statistical significance neither in axial length nor ocular blood flow.

**Fig. 6:**
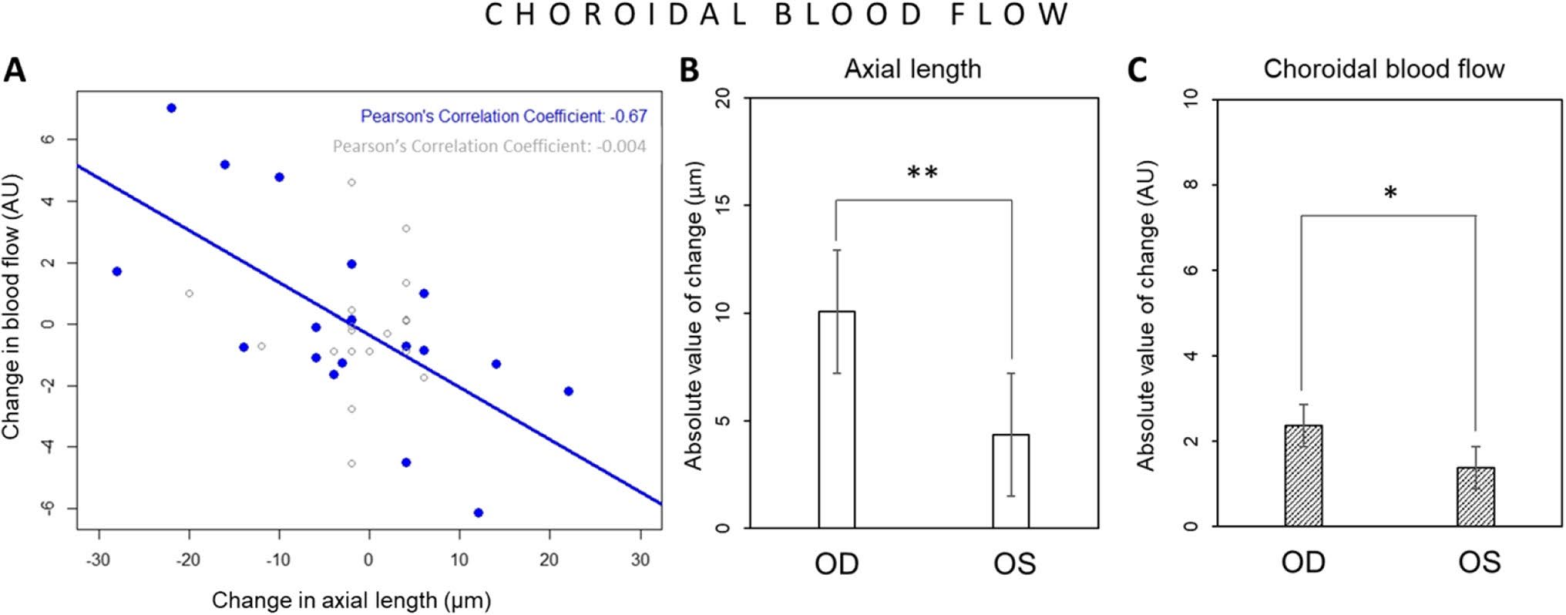
Effect of imposed positive defocus on choroidal blood flow. (**A**) Change in axial length was significantly correlated with change in choroidal blood flow in defocused eyes (marked in blue: *R* =  − 0.67, *p* < 0.01) while control eyes did not show such correlation (marked in gray: *R* =  − 0.004, n.s.). The absolute value of the induced changes in axial length (**B**) and choroidal blood flow (**C**) were significantly higher in defocused eyes (OD) than in control eyes (OS). Error bars represent the standard error of the mean (SEM). *p < 0.05; **p < 0.01

**Fig. 7:**
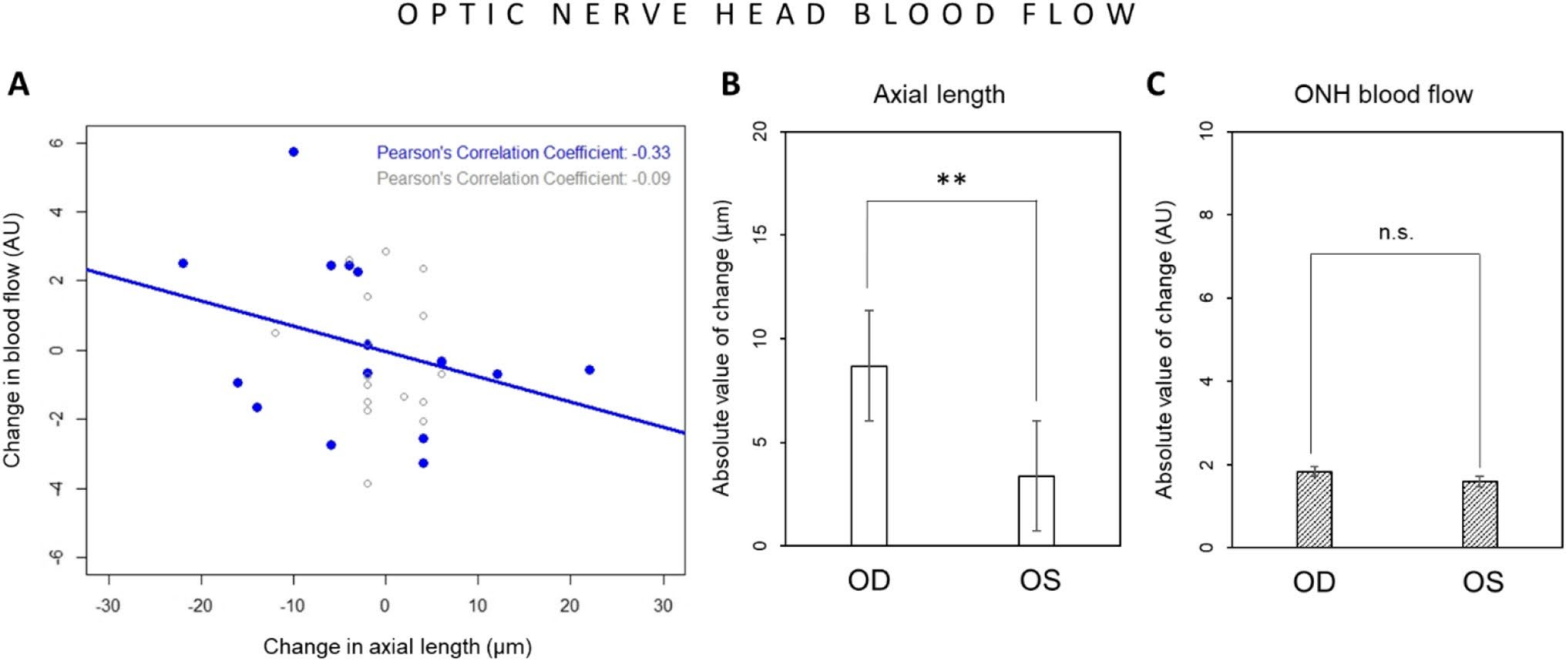
Effect of imposed positive defocus on optic nerve head (ONH) blood flow. (**A**) The changes in axial length were not correlated with the changes in ONH blood flow in defocused eyes (marked in blue: *R* =  − 0.33, n.s.) and in control eyes (marked in grey: R =  − 0.09, n.s.). (**B**) The absolute value of induced changes in axial length (**B**) was significantly higher in defocused eyes (OD) than in control eyes (OS), although absolute changes in ONH blood flow (**C**) were not significantly different between defocused and control eyes. Error bars represent the standard error of the means (SEM). **p < 0.01. n.s. not statistically significant

**Fig. 8:**
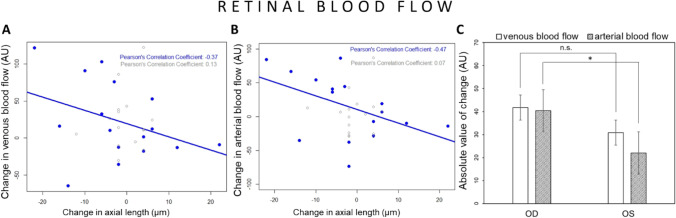
Effect of imposed positive defocus on retinal venular and arteriolar blood flow. (**A**) Changes in axial length were not correlated with changes in venular blood flow in defocused eyes (marked in blue: R =  − 0.37, *p* = 0.15) and in control eyes (marked in grey: *R* = 0.13, n.s.). (**B**) Changes in axial length showed a trend of correlation with changes in arteriolar blood flow in defocused eyes (*R* =  − 0.47, *p* = 0.06) with no correlation in control eyes (marked in grey: *R* = 0.07, n.s.). (**C**) The absolute value of induced changes in venular (white bars) and arteriolar blood flow (gray bars) were larger in defocused eyes (OD) than in control eyes (OS), although this reached a significance only in arterioles. Error bars represent the standard error of the mean (SEM). *p < 0.05. n.s. not statistically significant

Since myopes, emmetropes and hyperopes may respond to the positive defocus in different manner, as it has been shown before by Swiatczak and Schaeffel [17], data was presented as an absolute change of the analyzed variables in Figs. 6B, 6C, 7B, 7C, and 8B. Optical defocus (+ 2.5D) induced significant changes in axial length in exposed eyes, comparing with contralateral control eyes (n = 18, defocused eye: 10.06 ± 7.79 µm vs. control eye: 2.36 ± 2.16 µm, *p* < 0.01, Fig. 6B). Imposed defocus also induced changes in choroidal blood flow which were significantly greater in exposed eyes as compared to control contralateral eyes (n = 18, 2.35 ± 2.16 AU vs. 1.37 ± 1.44 AU, respectively, *p* < 0.05, Fig. 6C). Axial elongation occurred together with a decrease in choroidal blood flow and vice versa. Change in axial length after imposed defocus was significantly correlated with change in choroidal blood flow in defocused eyes (n = 18, R =  − 0.67, *p* < 0.01, Fig. 6A) while control untreated eyes did not show such correlation (n = 18, R =  − 0.004, n.s. Figure 6A).

Optic nerve head and retinal blood flow analysis included 16 participants. Positive defocus induced significant absolute change in axial length (n = 16, defocused eyes: 8.69 ± 6.66 µm vs. control eyes: 3.38 ± 2.70 µm, *p* < 0.01, Fig. 7B), but not in ONH blood flow (1.83 ± 1.47 AU vs. 1.59 ± 0.96 AU, n.s., respectively) (Fig. 7C). The changes in axial length were not correlated with the changes in ONH blood flow in defocused eyes (n = 16, R =  − 0.33, n.s., Fig. 7A) and in control untreated eyes (n = 16, *R* =  − 0.09, n.s., Fig. 7A).

From each LSFG scan centered at the ONH, one retinal arteriolar segment and one retinal venular segment in the peripapillary region were chosen for retinal blood flow assessment. Changes in retinal blood flow showed no correlation with changes in axial length in defocused eyes, although a trend of correlation was seen for arteriolar blood flow (*R* =  − 0.37, *p* = 0.15 and *R* =  − 0.47, *p* = 0.06 for venular and arteriolar retinal blood flow, respectively, Fig. 8A and 8B). Moreover, the absolute value of changes in retinal blood flow was significantly different between defocused and control eyes for retinal arterioles, but not venules (arterioles: defocused eyes: 40.36 ± 26.49 AU vs. control eyes: 21.99 ± 20.81 AU, *p* < 0.05, venules: defocused eyes: 41.77 ± 38.31 AU vs. control eyes: 30.83 ± 31.56 AU, *p* = 0.46, Fig. 8C).

## Discussion

The main goal of the current manuscript was to determine whether there is a negative correlation between changes in axial length and choroidal blood flow. This would suggest that eye elongation (resulting from thinning of the choroid) is accompanied with a decrease in choroidal blood flow while eye shortening (resulting from thickening of the choroid) involves an increase in choroidal blood flow. Our results show that this is, in fact, the case (Fig. 6A, showing a significant correlation between changes in axial length and changes in choroidal blood flow).

We found that changes in axial length, induced by monocularly imposed positive defocus, are negatively correlated with changes in choroidal blood flow, but not with changes in blood flow in the ONH or retina.

It has been shown before that axial length in myopic and emmetropic eyes of young adults may change in opposite directions when positive defocus is imposed [15, 17]. Except for one subject with − 4.25 D of myopia who responded like an emmetrope, most of the myopic subjects in the current study developed longer eyes.

### Choroidal blood flow

It has been shown before that axial length is negatively correlated with the thickness of the choroid, especially in myopic eyes [44]. Obviously, more myopic eyes develop thinner choroids. Furthermore, it was shown that changes in axial length induced by visual stimulation like optical defocus are significantly correlated with changes in subfoveal choroidal thickness in both human and animal models [10, 24, 25, 45, 46]. The current study shows that ocular response to imposed optical defocus is clearly correlated with changes in choroidal blood flow. There was a significant correlation between axial elongation and decreased choroidal blood flow. The finding is in line with previous studies which have shown that more myopic eyes have a larger blood flow deficit in the choroid and lower choroidal vascularity index [47, 48].

Along with a hypothesis that thinner choroid results from decreased blood flow, it can be assumed that less nutrients and oxygen are delivered to the outer retina and the sclera. Indeed, animal models and human studies have shown that myopia development is associated with hypoxic environment in the sclera [27, 49]. Moreover, anti-hypoxia drugs slowed the progression of experimental myopia in guinea pigs, as well as, prazosin, a vasodilator, was found to increase choroidal blood perfusion and inhibit myopia progression by increasing choroidal thickness and reducing scleral hypoxia [28, 49].

### ONH blood flow

ONH blood flow assessment includes measurements of both local disc circulation and large retinal blood vessels. Altered blood flow in optic disc was previously reported in glaucoma eyes [2, 50] and in ocular hypertension [3, 51]. In our study, the correlation between ONH blood flow change and altered axial length after positive defocus had similar trend as changes in choroidal blood flow, but it did not reach significance. Since retinal blood flow was not associated with altered axial length after positive defocus, that would explain why the signal obtained from the ONH was more diffuse and its correlation with change in axial length was weak.

### Retinal blood flow

Other studies have shown that retinal blood flow, vessel diameter and oxygen saturation are significantly decreased in high myopic eyes [4, 52]. In our study, we did not find a significant correlation between axial elongation and change in retinal blood flow, although defocused eyes had significantly greater change in arteriolar blood flow in the retina, compared to control eyes. Moreover, the correlation between changes in arteriolar blood flow and axial length barely failed to attain statistical significance. Thus, the signaling cascade leading to concurrent changes in axial length and choroidal blood flow showed a weak effect on retinal blood flow. If we were to assume the release of one or more vasoactive mediators locally in the choroid, this would show a weaker effect on retinal vessels because of the anatomical distance and the presence of the outer blood retinal barrier. The difference may also be due to the fact that highly myopic eyes may have developed morphological and functional changes in the fundus induced by the exceeded elongation of the eyeball, which may result in tissue remodeling and pathological changes in vascularity. However, our experiments were focused on short-term changes in ocular blood flow induced by defocus and included only healthy subjects with mild or moderate refractive errors. It has also been shown before that retinal blood flow is very sensitive to retinal activity levels. Visual stimulation of the retina by full-field flickering light strongly increased blood flow velocity in the central retinal artery and vein in normal eyes [7]. Moreover, the individual increase in retinal blood flow during flicker stimulation varies greatly between subjects [8] which may be a reason for the weak correlation between changes in axial length and changes in retinal blood flow in our study. Lastly, choroidal blood flow and axial length measurements were colocalized in the central macular region, whereas retinal blood flow was measured in the peripapillary region, and it may be that the effects of the signaling cascade induced by the positive defocus are not uniform at different sites in the ocular fundus. It has also been reported that the increase in volumetric retinal blood flow upon flicker-light stimulation is greater in arteries than in veins [53], which was also observed in our study as a significant difference in arterial, but not venous blood flow between defocused and control eyes induced after imposed positive defocus.

It has been shown that the amount of anisometric myopia is correlated with an ocular dominance [54]. Cheng et al. have shown that dominant eyes develop more myopia in anisometropic eyes. However, this correlation was found significant only when anisometric myopia exceeded − 1.75 D. In our current study, we did not record data on ocular dominance. Therefore, we cannot fully rule out a potential bias introduced by ocular dominance. On the other hand, we had excluded subjects with anisometropia or astigmatism of more than 1D and there was no correlation between ocular dominance and myopia in cases of low anisometropia [54]. Second, our study design defined right eyes as experimental eyes and left eyes as control. Therefore, any potential bias introduced by ocular dominance was also “randomized”.

## Conclusions

Our study showed that the retinal signaling cascade, activated by imposed positive defocus, targets selectively choroidal blood circulation, and does not significantly influence optic nerve head and retinal blood flow after short-term visual exposure. We speculate that there is a highly specific retinal mechanism for eye growth inhibition that is activated by imposed positive defocus and triggers an increase in choroidal blood flow which, in turn, can optimize energy/oxygen supply to the retina and sclera. Such a mechanism is likely to be very important in early phases of myopia development in children.
